# The Lumbodorsal Fascia as a Potential Source of Low Back Pain: A Narrative Review

**DOI:** 10.1155/2017/5349620

**Published:** 2017-05-11

**Authors:** Jan Wilke, Robert Schleip, Werner Klingler, Carla Stecco

**Affiliations:** ^1^Department of Sports Medicine, Goethe University Frankfurt, Frankfurt am Main, Germany; ^2^Fascia Research Group, Neurosurgical Clinic Guenzburg, Ulm University, Ulm, Germany; ^3^Department of Molecular Medicine, Institute of Human Anatomy, University of Padova, Padova, Italy

## Abstract

The lumbodorsal fascia (LF) has been proposed to represent a possible source of idiopathic low back pain. In fact, histological studies have demonstrated the presence of nociceptive free nerve endings within the LF, which, furthermore, appear to exhibit morphological changes in patients with chronic low back pain. However, it is unclear how these characteristics relate to the aetiology of the pain. In vivo elicitation of back pain via experimental stimulation of the LF suggests that dorsal horn neurons react by increasing their excitability. Such sensitization of fascia-related dorsal horn neurons, in turn, could be related to microinjuries and/or inflammation in the LF. Despite available data point towards a significant role of the LF in low back pain, further studies are needed to better understand the involved neurophysiological dynamics.

## 1. Introduction

Disc pathologies, as diagnosed using magnetic resonance imaging (MRI), do not necessarily represent the causal substrate of low back pain [1, 2]. Hence, a variety of predictors including psychological, environmental, genetic, or other morphological factors have been discussed [3]. Concerning the latter, Panjabi [4] proposed that microinjuries in lumbar connective tissues may be a contributing factor in idiopathic low back pain. Although his hypothesis referred to paraspinal connective tissues only, other authors argued that the lumbodorsal fascia (LF) should also be considered as a candidate for similar microinjuries [5, 6]. During recent years, a plethora of studies have been published connected to this novel hypothesis. While some of them appear to suggest a potential nociceptive capacity of the LF, the clinical relevance of these indications for a better understanding and treatment of low back pain remains to be elucidated. The present paper therefore aimed to delineate the role of the LF in patients with low back pain, with special focus on combining findings from histological studies and experimental research.

## 2. Method of Investigation

A thorough review analyzing current literature on three topics was conducted: (1) histological evidence for a potential nociceptive innervation of the LF, (2) morphological differences of the LF between low back pain patients and healthy subjects, and (3) nociceptive and nociception-related responses of the LF to experimental irritation. Studies published in PubMed, ScienceDirect, and Google Scholar until September 2016 were included. They were critically evaluated for their potential support (or lack of support) concerning the hypothesis that the LF could be a causal factor for low back pain.

## 3. Results and Discussion

### 3.1. Morphological Alterations

Several cases of a clear macroscopic hernia in the LF have been described [7–10]. However, all these reports agreed that such obvious cases are most likely rare exceptions representing a small minority of low back pain patients only. Dittrich [11, 12] examined the posterior layer of the LF—as well as histological sections taken from the tissue—during low back surgery. While he did not describe the number of patients examined, he reported the frequent finding of signs of injury and/or repair in this tissue and supported this by means of photographic documentation. Bednar et al. [13] examined the histology of samples from the posterior layer of the LF, which had been obtained during lumbar surgery from 24 patients suffering from low back pain. The included patients had not undergone previous lumbar surgery. Light and electron microscopy of the tissue samples revealed frequent microscopic changes suggestive of ischemia or inflammatory processes. However, since no control group was included in the study, it remains to be elucidated if similar abnormalities occur in asymptomatic persons also.

Langevin et al. [6] compared the mechanical behaviour of the posterior layer of the LF in chronic low back pain patients and age-matched, healthy controls. Using ultrasound recordings, the authors examined the shear-motion within the posterior layer of the LF during passive lumbar flexion movements. Compared to the controls, the low back pain group exhibited a significant reduction in shear-strain of about 20%. Moreover, a large share of the screened patients displayed an increased thickness of this fascial layer, albeit the difference in thickness was found to be significant in male patients only.

### 3.2. Innervation

Various histological examinations have documented the presence of unmyelinated terminal nerves in the LF (Table 1). The identified nerves include such with a presumably nociceptive potential (i.e., positive for CGRP staining) as well as such that clearly possess a nociceptive capacity (i.e., positive for SP staining). Interestingly, a study investigating the distribution and density of CGRP-positive fibers in different tissues reported a three times higher density in the LF than in the spinal muscles [14]. Furthermore, the density of nociceptive fibers was found to be increased in the inner layer of the rat LF, following chronic inflammation induced by Complete Freund's Adjuvant [15].

### 3.3. Experimental In Vivo Studies

Several trials have applied noxious stimuli to the posterior layer of the LF or other fasciae in order to elicit nociceptive responses under in vivo conditions. Available studies can be categorized into three groups being based on the use of (1) mechanical, (2) chemical, or (3) electrical stimulation.

Using a sharpened watchmaker's forceps, Pedersen et al. [16] mechanically pinched the LF of decerebrated cats and were able to trigger spastic contractions of the back muscles (in most cases ipsilateral), as well as of the hamstring and gluteal muscles (ipsilateral leg). Compared to pinching the underlying muscle tissues, the observed reactions were much more pronounced in response to pinching the fascia. A recent experiment done by Taguchi and colleagues [17] revealed, moreover, that pinching the posterior layer of the rat LF and irritating it by means of a chemical substance (hypertonic saline) induce clear responses in a substantial number of neurons of the spinal cord dorsal horn. Since applying hypertonic saline is considered to represent an effective stimulus for type VI afferents, the authors interpreted their findings as evidence for the nociceptive functional capacity of the LF. The study demonstrated furthermore that causing a chronic inflammation in the local musculature induces a threefold increase of dorsal horn neurons, which in turn respond to stimulation of the posterior layer of the LF. In another trial, Taguchi et al. [18] pinched the rat crural fascia and found an increased expression of c-FOS, a marker of neural activation induced by tissue injury and nociceptive stimulation, in the spinal dorsal horn. The number of identified nuclei was largest in the segments L2 to L4, peaking at L3. Here, c-FOS expression was about 2.5 times higher when compared to a sham stimulus (cutting the skin only).

Besides the works of Taguchi and colleagues, two other studies have examined nociceptive responses to fascial stimulation with hypertonic saline. In their animal study with rats, Gibson et al. [19] examined injection-provoked changes of pain sensitivity after induction of delayed onset muscle soreness (DOMS) in the lower limb. While hypertonic saline injected to the investing fascia provoked considerable pain, no comparable response was observed when the substance was applied into the muscle itself or the nonexercised muscle of the contralateral leg. As microinjuries and inflammation are suspected to be a major cause of pain in DOMS [20], the observations indicate a high nociceptive susceptibility of fascia to these processes. Although the data of Gibson and colleagues were collected for the lower limb, it might be inferred that excessive loading leading to inflammation and microfailure induces pain responses in the LF, too. This hypothesis is corroborated by two recent studies. In a trial with humans, Schilder et al. [21] demonstrated that chemical stimulation of the LF via hypertonic saline tends to induce a longer lasting (~15 versus ~10 minutes) and more intense pain perception than injection into related muscular tissues. Interestingly, only the injection into the fascia provoked affective pain descriptions (e.g., agonizing, heavy, and killing) that are often reported by patients with low back pain. Albeit not using hypertonic saline, Deising et al. [22] injected the nerve growth factor into the fascia of the erector spinae muscles at the lumbar level. They observed a long-lasting sensitization to mechanical pressure (days 1 to 7) and to chemical stimulation by means of acidic solution (up to two weeks).

With regard to electrical stimulation, the available evidence suggests that the LF is also responsive to this pathway of irritation. After induction of delayed onset muscle soreness in the elbow flexors, pain thresholds of the fascia decrease significantly more than those of the underlying muscle tissue [23]. Similar to the study of Gibson et al. [19], this might indicate substantial nociceptive responses of the connective tissue to presence of inflammatory processes, respectively, microinjury. In addition to the tissue of the upper limb, also the LF generates pain sensations upon electrical stimulation, which appear to be more prominent when compared to muscular tissue. The electrical pain threshold of the LF (3.02 ± 1.92 mA) was shown to be considerably lower than that of the erector spinae muscle (8.54 ± 5.57). Moreover, analogous to the findings concerning chemical stimulation, the controlled electrical irritation of fascial low back tissue leads to stronger pain reactions than the stimulation of the lumbar muscles [24].

## 4. Conclusions

The LF of both rodents and humans displays a dense innervation with nociceptive afferents. In addition, chemical stimulation of the LF has been shown to elicit severe and particularly long-lasting sensitization processes. These innervations-related studies indicate that the LF exhibits a clear nociceptive neural capacity and therefore may be a source of pain in some cases of low back pain.

Concerning morphological changes, the ultrasound examination of Langevin et al. [6] demonstrated a reduction in shear-strain transmission in the LF of chronic low back pain patients compared with healthy controls. It seems plausible that this change could be explained by tissue adhesions induced by previous injury or inflammation which would be in accordance with the aetiology proposed by Dittrich [12] and Bednar et al. [13]. In addition to this, immobility or inactivity represents another factor potentially causing decreased shear-strain due to the thixotropic behaviour of hyaluronic acid between the layers [33]. Hence, it is well possible that the tissue alterations represent the result of a reduction in everyday lumbar movements in low back pain patients. Nonetheless, these findings cannot answer the question whether the observed tissue changes are a cause or a consequence of low back pain.

Several in vivo examinations indicate that the nervous system seems to respond with a particularly strong and long-lasting sensitization of dorsal horn neurons towards mechanical, chemical, and electrical stimulation of the LF. Assuming a proneness to microinjuries, overloading, and/or inflammation, it might be inferred that such tissue irritations could trigger substantial nociceptive adaptations which are frequently observed in patients with idiopathic back pain.

**Figure 1:**
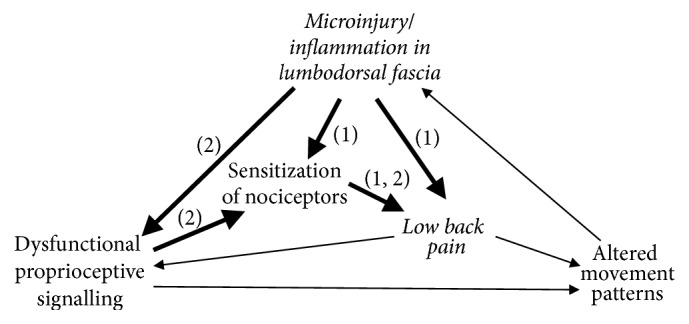
The current literature supports a potential nociceptive function of LF in the aetiology of low back pain. This graph represents two of several possible scenarios in respective cases of fascia generated low back pain. (1) Microinjuries and/or inflammation and resulting irritation of nociceptive nerve endings in lumbar fascia may directly induce back pain, accompanied with a sensitization of fascial nociceptors. In a second pathway (2) tissue deformation due to injury and/or immobility may impair proprioceptive signalling. This induces a sensitization of fascial nociceptors wide, which then alters the functioning of related polymodal neurons in the spinal cord to respond more strongly to potential nociceptive signalling, even to gentle stimulation. Combinations of both pathways are of course also possible. Figure partially based on Langevin & Sherman [25].

**Figure 2:**
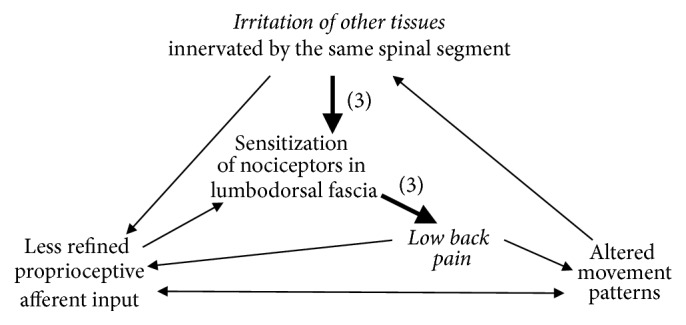
In a third scenario for fascia generated low back pain (3) irritation of other tissues—such as muscle fibers, facet joint capsules, spinal nerve roots, or the annulus fibrosus of the discs—could elicit an increased sensitivity in the LF innervated by the same segment of the spinal cord. The increased sensitivity of fascial nerve endings would then lead to nociceptive signalling, even in response to gentle stimulation. A combination with the pathways described in Figure 1 is also possible. Figure partially based on Langevin & Sherman [25].

Taken together, these findings suggest that the LF, besides other frequently suspected structures such as the intervertebral disk, also represents a potential pain generator in patients with lumbar disorders. Three different mechanisms for fascia-mediated low back pain sensations can be distinguished: (1) microinjuries irritating nociceptive nerve endings in the LF may directly induce back pain; (2) tissue restructuration, for example, following microinjury, immobility, or chronic overloading, may compromise proprioceptive signalling, which by itself could decrease the pain threshold by means of an activity-dependent sensitization of wide dynamic range neurons [34]; and, finally, (3) nociceptive input from other tissues innervated by the same spinal segment could elicit an increased sensitivity in the LF. In addition to these theories, various combinations of three processes may be possible (Figures 1 and 2).

The present review focused on the role of the lumbar fascia in idiopathic low back pain. Although a plethora of studies suggest a significant role of the LF in this large subgroup of patients, the relevance in specific disorders remains controversial. Kuslich et al. [35] used progressive local anaesthesia and mechanically stimulated each successive tissue layer during disc surgery in low back pain patients. While mechanical stimulation of the compressed nerve root induced strong radiating back pain symptoms, the same stimulation on the posterior layer of the LF failed to elicit similar responses in the majority of patients: local pain without radiation occurred in only 32 out of 193 patients. On the other hand, a sagging LF (bulges in the parasagittal plane identified using magnetic resonance imaging) has been shown to be correlated with adjacent lumbar segment disease [36]. Yet, it is unclear if this observation predisposes for the pathology or vice versa.

**Figure 3:**
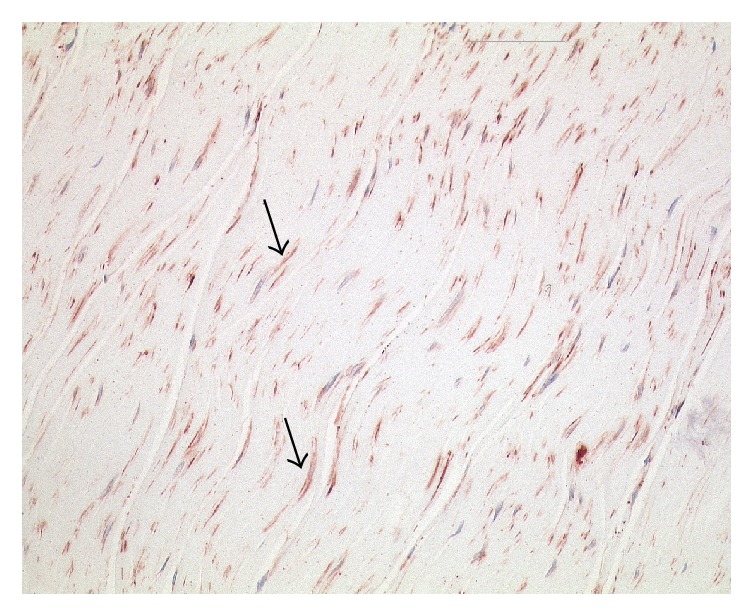
Example of a histological section taken of the posterior layer of LF at the level of L2. Arrows indicate fibers containing alpha-smooth muscle actin, an immunohistochemical marker for myofibroblasts, which is stained in red. Nuclei are stained dark blue. Although nothing is known about the presence of low back pain in this donor, the high density of myofibroblasts in this tissue is notable and is reminiscent of comparable histological sections in fascial pathologies such as Frozen Shoulder (Bunker et al. 1995). A high density of these contractile cells is usually only seen in fibrotic pathologies and/or in tissue conditions with an increased injury repair activity. Length of image 225 *μ*m.

Notwithstanding, the question of how often any of the aforementioned, fascia-related aetiologies manifest in idiopathic low back pain patients provides an important and challenging background for future investigation. The clarification of this question promises to offer valuable contributions for the treatment and prevention of low back pain. Future investigations could include histological examinations (see example in Figure 3, from our laboratory) as well as high-resolution ultrasound [37] and MRI investigations of the LF in back pain patients.

## Figures and Tables

**Table 1:** Histological studies exploring the potential nociceptive innervation of the posterior layer of the LF.

Study	Tissue source	Method	Nerve endings found	Remarks
Stilwell [26]	Macaca mulatta (*n* = 17), rabbit (*n* = 4)	Methylene blue	Rich supply by FNE. Groups of large Pacinian corpuscles at penetration points of dorsal rami through the fascia. Also small Pacinian-like and Golgi-Mazzoni corpuscles.^*∗*^	Study included human tissues too. However, no nerve type analysis was performed on those.

Hirsch [27]	Human (*n* = ?)	Methylene blue	FNE, “complex unencapsulated endings” ^*∗*^.	Number of donors not mentioned. Also found: unmyelinated nerve fiber network associated with blood vessels

Yahia et al. [28]	Human (*n* = 7)	IH: neurofilament protein and S-1 00 protein	FNE, Ruffini, Pacini.^*∗*^	

Bednar et al. [13]	Human (12),	IH: neuron-specific enolase	No terminal nerves found.^*∗*^	Study performed with CLBP patients only. Found: small peripheral nerve bundles at the margins and in association with small vessels.

Corey et al. [29]	Rats (5)	3D reconstructions of thick (30–80 *µ*m) tissue sections IH: PGP 9.5, CGRP, fast blue	CGRP-positive FNE.	Also found: Some nonterminating CGRP-labeled fibers along blood vessels.

Tesarz et al. [30]	Rat (*n* = 8) Human (*n* = 3)	IH: PGP 9.5, TH, CGRP, SP	Rich innervation with presumable nociceptive nerve endings (PG, CGRP).	Most nerve fibers located in the outer layer of the lumbar fascia and in the subcutaneous connective tissue.

Benetazzo et al. [31]	Human (2)	3D reconstruction of serial sections IH: S100	Study did not investigate nerve terminations.	Small nerves (mean diameter 15 *µ*m) found, flowing from the superficial sublayer into the adjacent subcutaneous loose connective tissue. No nerves visible in intermediate and deep sublayers.

Hoheisel et al. [15]	Rats (10)	IH: PGP 9.5, TH, CGRP, SP	Rich innervation with presumable nociceptive nerve endings (SP, CGRP).	Inflammation of the fascia induced an increase of presumably nociceptive fibers.

Barry et al. [14]	Mice (4–8)	IH: PGP 9.5, CGRP, SP. Plus retrograde tracing.	Most nerve fibers contained CGRP	Two major subpopulations of neurons were found: those containing CGRP & SP and those containing CGRP but not SP. Innervation density was 3x higher in the thoracolumbar fascia than in muscles of the back

Mense and Hoheisel [32]	Rats (5)	IH: PGP 9.5, TH, CGRP, SP, TRPV1	Rich innervation with presumable nociceptive nerve endings (SP, CGRP, and TRPV1).	Inflammation of the fascia induced an increase of presumably nociceptive fibers.

IH: immunohistochemical analysis. FNE: free nerve endings. PGP 9,5: a universal marker for neural structures. TH: marker for sympathetic neurons. CGRP: marker for presumably nociceptive fibers. SP: marker for clear nociceptive fibers (containing substance P). TRPV1: a novel marker for transient receptor potential receptor subtype V1 (one of the main receptor molecules in the membrane of nociceptors). ^*∗*^Method of identification of termination of small nerves not mentioned. Not included in this table are studies on supraspinous, interspinous, or iliolumbar ligaments.
